# CSA-SA-CRTNN: A Dual-Stream Adaptive Convolutional Cyclic Hybrid Network Combining Attention Mechanisms for EEG Emotion Recognition

**DOI:** 10.3390/brainsci14080817

**Published:** 2024-08-15

**Authors:** Ren Qian, Xin Xiong, Jianhua Zhou, Hongde Yu, Kaiwen Sha

**Affiliations:** Faculty of Information Engineering and Automation, Kunming University of Science and Technology, Kunming 650500, China; qianren@stu.kust.edu.cn (R.Q.); xiongxin@kust.edu.cn (X.X.); hmgdd@stu.kust.edu.cn (H.Y.); shakaiwen@stu.kust.edu.cn (K.S.)

**Keywords:** EEG, emotion recognition, attention mechanism, dual-stream model, adaptive, hybrid network

## Abstract

In recent years, EEG-based emotion recognition technology has made progress, but there are still problems of low model efficiency and loss of emotional information, and there is still room for improvement in recognition accuracy. To fully utilize EEG’s emotional information and improve recognition accuracy while reducing computational costs, this paper proposes a Convolutional-Recurrent Hybrid Network with a dual-stream adaptive approach and an attention mechanism (CSA-SA-CRTNN). Firstly, the model utilizes a CSAM module to assign corresponding weights to EEG channels. Then, an adaptive dual-stream convolutional-recurrent network (SA-CRNN and MHSA-CRNN) is applied to extract local spatial-temporal features. After that, the extracted local features are concatenated and fed into a temporal convolutional network with a multi-head self-attention mechanism (MHSA-TCN) to capture global information. Finally, the extracted EEG information is used for emotion classification. We conducted binary and ternary classification experiments on the DEAP dataset, achieving 99.26% and 99.15% accuracy for arousal and valence in binary classification and 97.69% and 98.05% in ternary classification, and on the SEED dataset, we achieved an accuracy of 98.63%, surpassing relevant algorithms. Additionally, the model’s efficiency is significantly higher than other models, achieving better accuracy with lower resource consumption.

## 1. Introduction

Emotion is a complex state influenced by various feelings, behaviors, and thoughts when the human brain is stimulated [1]. It plays a crucial role in shaping our mental states and guiding behavioral decisions. Positive emotions are beneficial to human health and attitude, while negative emotions may have adverse effects on mental health and even cause serious psychological problems [2]. Accurate identification of emotions can help people understand a person’s psychological state, thus making corresponding adjustments and avoiding negative impacts caused by emotional states, such as depression, negative transformation of thinking and emotion, and emotional numbness [3]. Currently, the advancement of emotion recognition systems has become a prominent focus across various disciplines, including psychology, artificial intelligence, computer vision, consumer behavior analysis, medical treatment, and other related fields [4].

Understanding human emotions is a complex task that involves analyzing both non-physiological and physiological signals. Facial expressions, voice tone, and text messages are some of the non-physiological signals we use to decipher someone’s emotions [5,6]. On the other hand, physiological signals like brain activity, muscle movements, and skin responses provide a deeper insight into our emotional state [7]. People can mask their true emotions, making it tricky to accurately predict how their emotions solely based on non-physiological signals they emit. In contrast, physiological signals like EEG offer a more reliable glimpse into someone’s emotions. EEG stands out as a star performer in capturing emotions in real-time with high precision and at a reasonable cost. It has become a go-to method for reading emotions without invading someone’s privacy [8,9,10].

In the early stages of EEG emotion recognition research, scholars focused on traditional EEG features and used algorithms like Support Vector Machines (SVM) and K-nearest neighbors (KNN) [11,12]. However, manual feature selection in machine learning is time-consuming and laborious, and the accuracy is not ideal. With the advancement of technology, deep learning has become a new solution for EEG emotion recognition, demonstrating superior performance in identifying human emotions [2,13,14]. Various models, including convolutional neural networks(CNN) [15], capsule networks(CapsNet) [16], and long short-term memory networks(LSTM) [17], have been developed by researchers to comprehend human emotions through EEG signals. For instance, Huang et al. [18] devised an S-EEGNet model, achieving an accuracy of 89.91% and 88.31% on the DEAP dataset. In a separate study, Zheng and colleagues [19] introduced a CNNFF architecture with an average classification accuracy of 93.61% for valence and 94.04% for arousal, respectively. Although the above studies have achieved good results, they do not provide further results. Zheng et al. [20] trained a DBN to analyze differential entropy features across multiple EEG channels, comparing deep and shallow models using the SEED dataset with an average accuracy of 86.08%, 83.99%, 82.70%, and 72.60% in DBN, SVM, LR, and KNN, respectively. Cui and his team [21] developed a new model called RACNN for classifying emotions in EEG data, achieving over 95% identification accuracy on both valence and arousal classification tasks using DEAP and DREAMER datasets. However, these methods only use a single network model, and the recognition accuracy needs to be improved. To further improve the accuracy of EEG emotion recognition, researchers have been experimenting with hybrid network models that combine techniques like CNN, RNN, LSTM, GCN, and attention mechanisms in innovative ways. Zong et al. [22] introduced the FCAN-XGBoost algorithm, which blends FCAN and XGBoost algorithms for processing DEAP and dream datasets with accuracies of 95.26% and 94.05%, respectively. Chakravarthi et al. [23] developed an automated system using a combination of CNN and LSTM based on the ResNet-152 algorithm, achieving an impressive accuracy of 98%. 

The aforementioned methods have yielded a series of satisfactory results. However, the sheer volume of irrelevant information present in EEG signals means that analyzing them requires much computational power [24]. Cutting through this noise to uncover the true emotional cues is a challenge that researchers are striving to overcome. Also, the current methods for extracting features often result in a significant loss of important information [23], presenting a pressing issue that needs to be addressed promptly.

To address the aforementioned issues, a two-flow adaptive convolutional cyclic mixing network (CSA-SA-CRTNN) combined with an attention mechanism is proposed for emotion recognition in EEG. The model initially utilizes the CSAM module to assign weights to EEG channels and then inputs the EEG signals into the two-stream adaptive convolutional circulation network to extract local spatiotemporal features. Subsequently, the local features extracted from the two-stream network are spliced and inputted into the time-series Convolutional network combined with a multi-head self-attention mechanism (MHSA-TCN) to extract global information. Finally, the feature vectors are fed into the fully connected layer, and a softmax classifier is employed for emotion classification.

The CSA-SA-CRTNN model proposes a novel Channel Selection Attention Mechanism (CSAM) to selectively focus on multi-channel EEG signals and incorporates a newly devised Multi-Head Self-Attention Mechanism (MHSA) with various feature extraction networks. This approach enables the model to attend to emotion-related critical information, significantly reducing the impact of redundant information and enhancing model efficiency compared to existing emotion recognition studies that utilize full-channel EEG signals. Furthermore, we introduce an Adaptive Convolutional Recurrent Neural Network (SA-CRNN), a pioneering network architecture that can automatically adjust the window size for feature extraction, effectively minimizing information loss.

In summary, the contributions of this paper are as follows:

A novel approach has been developed for attending to various brain signals, referred to as the channel-wise attention module (CSAM). This module function selectively highlights the most pertinent aspects of the signal while disregarding extraneous information that is inconsequential about emotions. This facilitates more efficient cognitive processing, conserving time and energy resources while ensuring focused attention on critical informational components.

A multi-head self-attention mechanism (MHSA) has been developed and integrated with CRNN and TCN to effectively capture both local and global key features in emotional data. This innovative technique significantly enhances our model’s capacity for accurate emotion recognition while also reducing computational complexity.

An adaptive convolutional recurrent network (SA-CRNN) is proposed for extracting local EEG information. This network can adaptively match appropriate convolutional stride and pooling parameters for the provided convolutional kernel size, effectively addressing the issue of information loss and improving the final recognition performance.

A dual-stream adaptive convolutional recurrent hybrid network with an attention mechanism (CSA-SA-CRTNN) is designed for emotion recognition. 

## 2. Related Work

This section reviews the current research status of EEG emotion recognition, focusing on the introduction of emotion models, feature extraction, deep learning, feature fusion and hybrid models, and attention mechanisms.

### 2.1. Emotional Model

**Figure 1:**
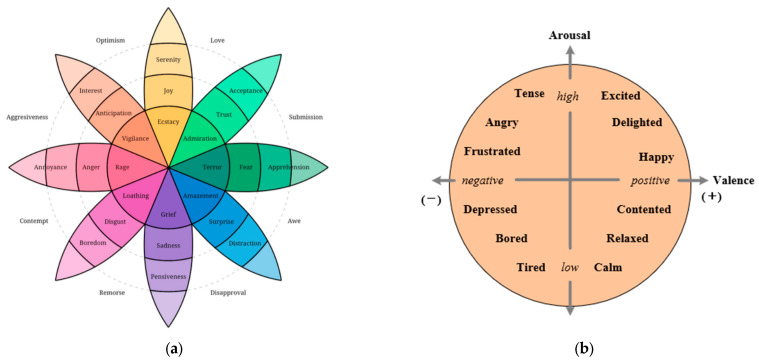
Emotion model: (**a**) discrete model, (**b**) two-dimensional valence–arousal model.

Researchers studying emotion recognition use two models to describe emotions: the Discrete Model [25,26] and the Dimensional Model [27,28]. The Discrete Model sees emotions as distinct points and believes that emotions are made up of basic emotions. Although the Discrete Model is straightforward to understand, it falls short in describing the complexity of human emotions, as shown in Figure 1a. The Dimensional Model tries to understand human emotions by mapping them in a two- or three-dimensional space. The Valence-Arousal Model is a classic dimensional model, which represents different emotions as points on a two-dimensional plane of Valence and Arousal, as shown in Figure 1b. Other emotions are made up of different combinations of Valence and Arousal [29]. The Dimensional Emotional Model is continuous and can express emotions across a wide range. 

### 2.2. Feature Extraction in EEG Emotion Recognition

In the process of emotion recognition utilizing EEG data, the primary objective of feature extraction is to obtain information that can effectively reflect an individual’s emotional state. Subsequently, this information can be utilized in emotion classification algorithms. Feature extraction serves as a pivotal step in emotion recognition tasks, as the ability to extract discriminative features influences the ultimate effectiveness of the recognition. There are four traditional methods for analyzing EEG features: time-domain analysis, frequency-domain analysis, time–frequency-domain analysis, and nonlinear feature analysis. Tripathi et al. [30] extracted nine features from the DEAP EEG signals, including mean, median, maximum, minimum, standard deviation, variance, range, skewness, and kurtosis. They employed two types of classification methods, Deep Neural Network (DNN) and Convolutional Neural Network (CNN) and achieved excellent results. Yuvaraj et al. [31] extracted statistical features, fractal features, Hjorth parameters, high-order spectral features, and wavelet coefficients from the DEAP dataset and used shallow classifiers for classification. The accuracy rates of valence and arousal were 78.18% and 79.90%, respectively. Nawaz et al. [32] used a feature selection algorithm (Principal Component Analysis, PCA) to compare power, entropy, fractal metrics, statistical features, and wavelet features, achieving an accuracy rate of 77.62% and 78.96% for valence and arousal, respectively. Zheng et al. [33] proposed an EEG emotion recognition method called ERHGCN based on a Hierarchical Graph Convolutional Network (HGCN). Firstly, six different features, including Power Spectral Density (PSD), Differential Entropy (DE), Differential Asymmetry (DASM), Rational Asymmetry (RASM), Asymmetry (ASM), and Differential Causality (DCAU) were extracted from five frequency bands. These extracted six features were then input into the HGCN model and integrated through two fully connected layers for emotion recognition.

### 2.3. Deep Learning-Based EEG Emotion Recognition

In recent years, deep learning methods have gradually become mainstream in the field of artificial intelligence. In the aspect of EEG emotion recognition, this method has also been widely favored by researchers. Unlike machine learning methods, deep learning methods can automatically extract signal features, greatly accelerating research in EEG emotion recognition. Algarni et al. [17] employed a stacked bidirectional long short-term memory (Bi-LSTM) model and conducted experiments on the DEAP dataset. They extracted statistical features, wavelet features, and Hurst exponents from the dataset, implemented feature selection using a binary grey wolf optimizer, and achieved average accuracies of 99.45%, 96.87%, and 99.68% for the arousal, valence, and liking dimensions, respectively. Zhong et al. [34] proposed a Regularized Graph Neural Network (RGNN) for EEG-based emotion recognition. This study modeled the inter-channel relationships in EEG signals through the adjacency matrix in graph neural networks. Additionally, two regularizers were proposed, namely Node Domain Adversarial Training (NodeDAT) and Emotion-aware Distribution Learning (EmotionDL), to better handle EEG variations and noisy labels across different subjects.

### 2.4. Feature Fusion and Hybrid Models in EEG Emotion Recognition

Recent studies [33,35] have revealed that combining multiple EEG features leads to superior results in detecting emotions compared to using just one feature. In the field of emotion recognition research using deep learning methods, feature fusion is often closely associated with the development of hybrid models [36,37,38]. For example, Feng et al. [39] proposed a new model called ST-GCLSTM, which combines SGCN and attention-enhanced bidirectional LSTM. This model can obtain the temporal patterns of brain signals and capture the different intensities of these signals at different times, achieving a recognition accuracy of 95.52%. Li et al. [40] proposed a BLSTM network model based on multimodal attention, which can learn the optimal temporal features of EEG signals with a recognition accuracy of 81.3%. Overall, hybrid network models incorporating CNN, RNN, LSTM, GCN, and attention mechanisms tend to yield good results in emotion recognition tasks.

### 2.5. Attention Mechanism in EEG Emotion Recognition

Humans have the remarkable ability to focus on specific elements of a scene while filtering out other distractions—a phenomenon known as the attention mechanism. In recent years, the integration of neural networks with attention mechanisms has revolutionized the field of emotion recognition, particularly in EEG-based applications [39,41]. By selectively filtering and enhancing crucial components of the input data, these attention mechanisms have significantly boosted the accuracy of emotion recognition systems. Researchers like Zhang et al. [41] have developed innovative deep-learning models that leverage attention mechanisms to extract key features from EEG signals, yielding impressive classification results in emotion recognition tasks, and the classification accuracy reaches 92.47%. Similarly, Lew et al. [42] have explored the use of attention mechanisms in conjunction with adversarial networks to capture complex spatiotemporal relationships within EEG data, ultimately mitigating domain shift issues, and the classification accuracy reaches 98.15%. 

In summary, there is still room for improvement in the accuracy of EEG-based emotion recognition. To this end, we propose a novel hybrid model, CSA-SA-CRTNN, aiming to improve the accuracy of EEG emotion recognition while minimizing computational costs. Different from previous work in this field, the proposed CSA-SA-CRTNN model is a new hybrid model for EEG emotion recognition that combines multiple attention mechanisms.

## 3. Methodology

**Figure 2:**
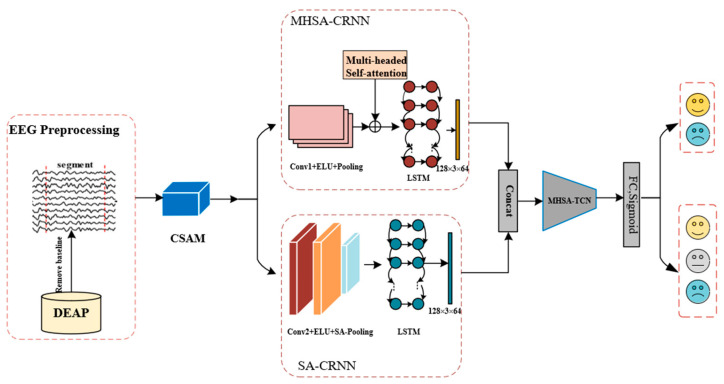
Frame diagram of the CSA-SA-CRTNN model. The model consists of four modules, namely the CSAM module, the SA-CRNN module, the MHSA-CRNN module, and the MHSA-TCN module.

The proposed CSA-SA-CRTNN model’s framework and process are displayed in Figure 2, which consists of four modules: CSAM, SA-CRNN, MHSA-CRNN, and MHSA-TCN. The CSAM assigns weights to EEG channels, while the dual-stream convolutional recurrent framework composed of SA-CRNN and MHSA-CRNN extracts local EEG features. The MHSA-TCN module extracts global EEG features, and the feature vectors are fed into the softmax classifier for emotion classification. In the following chapters, we will explain the model’s framework and process in detail.

### 3.1. Channel-Wise Spatial Attention Module(CSAM) 

**Figure 3:**
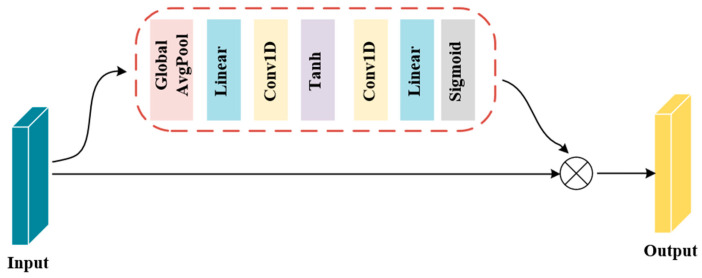
CSAM Structure Diagram.

Inspired by [43], a new channel attention mechanism (CSAM) has been introduced. It receives preprocessed EEG signals, allocates attention to channel dimensions, and enhances the effect of attention mechanisms on improving model performance. The proposed CSAM module can be found in Figure 3.

The module comprises a global pooling layer, linear layer, convolution layer, and activation layer. It allocates weights to the channel dimension, enhancing important information and minimizing unimportant information, thereby improving the ability to recognize and classify emotions while reducing computing costs. Table 1 shows the specific parameter settings for this module.

The CSAM first processes the input by applying average pooling. This step reduces the spatial dimensions while preserving the channel information. Following this, a linear layer (fully connected layer) is employed to project the channel information of the pooled vector into a higher-dimensional space, enabling the model to capture more complex relationships between channels. Subsequently, a convolutional layer is utilized to further extract features from this expanded feature space. The convolutional operation enhances the representational power of the network by learning local patterns within the channel-wise features. After the convolution, a tanh activation function is applied to introduce nonlinearity, allowing the model to learn complex decision boundaries. This nonlinearly transformed feature map then undergoes another linear transformation, designed to reshape it back to its original dimensions (prior to the initial average pooling), ensuring compatibility with the subsequent operations. Next, the Softmax function is applied along the channel dimension of the linearly transformed feature map. This step converts the feature values of each channel into a probability distribution, effectively assigning a weight (or importance) to each channel. The resulting attention map encodes the relative significance of each channel in the overall feature representation. Finally, this attention map is element-wise multiplied by the original input. This multiplication operation reinforces important channels while suppressing less significant ones, producing a weighted output that is more focused on salient features. In this manner, the CSAM module achieves weight allocation by emphasizing channels based on their individual contributions, enhancing the model’s ability to identify and utilize relevant information.

### 3.2. MHSA-CRNN

MHSA-CRNN comprises a Convolutional Recurrent Neural Network (CRNN) and a Multi-Head Self-Attention (MHSA) mechanism, as shown in Figure 2. It is capable of effectively extracting critical spatio-temporal features at a local level.

#### 3.2.1. CRNN

CRNN is the basic framework of the MHSA-CRNN module. To reduce the computational complexity of the model, a three-layer CNN and a two-layer LSTM are used here.

This module first uses CNN to extract spatial dimension information and then adopts LSTM to extract the temporal dependency of the signal, as shown in Equation (1):

(1) $$v_{st}=LSTM(Conv\left(x\right))$$

Here, x is the input signal, and $v_{st}$ is the feature vector output through the CRNN network. The structural composition and parameter settings of CRNN are shown in Table 2.

CNN consists of different layers, including a convolutional layer, an activation layer using exponential linear units (ELUs), and a maximum pooling layer. In the activation after convolution, the ELU function is preferred over the commonly used ReLU function. The kernel size and step size of the convolution operation are set to (32,40) and 1, respectively, using the same Settings as in the literature [43]. The pooling layer uses a kernel size of (1,75) with a stride length of 10. Next comes the LSTM model, which consists of two stacked layers. Each layer of the LSTM receives hidden states as input from the previous layer to learn more advanced feature representations in the data. While adding more layers can improve a model’s performance, it also increases training time and computational requirements. The input dimension of the LSTM unit is set to 80, and the hidden layer dimension to 64.

#### 3.2.2. Multi-Headed Self-Attention (MHSA)

**Figure 4:**
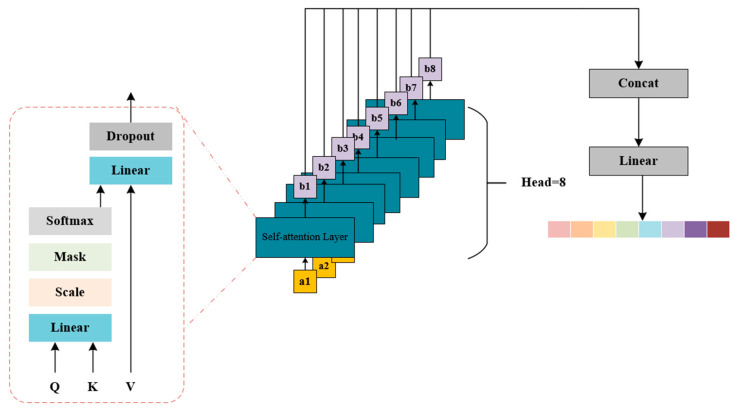
MHSA Structure Diagram.

To better understand contextual information and further improve model performance, we developed a novel multi-head self-attention mechanism, MHSA, and integrated it into CRNN to automatically capture dependencies among sequences and enhance the representation ability of the network. The structure of MHSA is shown in Figure 4.

The MHSA model utilizes multiple sets of query, key, and value mappings to concurrently compute several attention representations. These representations are then linearly stacked to produce the final multi-head attention representation. This approach allows the model to focus on different aspects of the input sequence, thereby enhancing the model’s ability to generalize.

In this paper, the Head of MHSA is equal to 8, and the overall calculation process is as follows:

(2) $$SA\left(Q,K,V\right)=softmax\left(\frac{{QK}^{T}}{\sqrt{d_{k}}}\right)V$$

(3) $$h_{i}=SA(QW_{i}^{Q},KW_{i}^{K},VW_{i}^{V})$$

(4) $$MHSA\left(Q,K,V\right)=Concat(h_{1},\ldots \ldots ,h_{8})W^{O}$$

Here, $SA\left(Q,K,V\right)$ represents the self-attention output, $d_{k}$ is the scaling factor, $W_{i}^{Q},$ $W_{i}^{K},W_{i}^{V}$ are the query, key, and value transformation matrix of the i_th head, respectively, $h_{i\;}$ represents the i_th head attention output, $MHSA\left(Q,K,V\right)$ is the final output of MHSA, and $W^{O}$ represents the transformation matrix of the output.

The self-attention mechanism acts as the central processing unit of the Multi-Head Attention (MHSA) algorithm, ensuring seamless operation. Initially, input features undergo linear transformations to yield query (Q), key (K), and value (V) features. These transformed features are then partitioned into three groups: Q, K, and V. The attention scores are computed by multiplying the query and key together and incorporating a scaling factor (Scale). Subsequently, these scores are adjusted based on an input mask to filter out irrelevant information. Following this, the attention scores undergo a Softmax function for normalization purposes. Ultimately, the output is generated by combining values with attention scores. This output then undergoes further transformation to revert to its original form, along with some Dropout processing for additional fine-tuning.

### 3.3. SA-CRNN

The structure of the Self-Adaptive Convolutional Recurrent Neural Network (SA-CRNN) is similar to the CRNN described in Section 3.2.1, as shown in Figure 2. This module is made up of a series of a three-layer CNN and a two-layer LSTM. It first extracts spatial dimension information through the Convolutional Neural Network (CNN) and then utilizes LSTM to extract the temporal dependency of signals. The CNN includes convolutional layers, ELU activation layers, and max pooling layers. Moreover, the convolutional stride, pooling kernel size, and pooling stride of the CNN can be adaptively adjusted according to the given convolutional kernel. This structural design aims to fully extract EEG emotional information and avoid the loss of feature information due to fixed parameter settings. The calculation methods for the convolutional stride and pooling layer parameters of the convolution operation in this module are as follows:

(5) $$\;\;\;\;\;\;\;\;s_{c}=\frac{k_{c}[-1]}{10}$$

(6) $$k_{p}=(1,\frac{k_{c}\left[-1\right]}{2})$$

(7) $$s_{p}=\frac{k_{c}[-1]}{10}$$

where $s_{c}$ is the convolutional stride, $k_{c}$[−1] is the element of the last dimension of the convolutional kernel, $k_{p}$ is the size of the pooling kernel, and $s_{p}$ is the pooling stride. In this module, the input dimension of the LSTM unit is 720, and the hidden layer dimension is 64.

### 3.4. MHSA-TCN

A common way to understand time-dependent problems is to use recurrent neural networks (RNNS). However, RNNS also has its disadvantages, such as difficulty in parallelizing calculations, low processing efficiency, and the risk of gradient explosion and gradient disappearance. To address these challenges, Bai S and their team [44] introduced temporal convolutional networks (TCN). TCN uses one-dimensional convolution, extended convolution, causal convolution, and residual convolution to maximize the ability of convolution to analyze time series. This approach is superior to LSTM (a type of RNN) in many different tasks. 

TCN is a technology that enables efficient parallel computing and is well-suited for processing large amounts of data. Its benefits include the ability to extract features of different scales by stacking multiple convolutional layers, accurately capturing local dependencies in sequence data by increasing the receptive field of the convolutional kernel and avoiding issues with gradient vanishing and explosion, etc. The main reason why TCN is so useful is that it significantly increases the receptive field [45]. The calculation method of the receptive field for the  i_th layer is as follows:

(8) $$F_{i}=\left(k-1\right)\times (\sum_{i=0}^{L-1}d_{i})+k$$

where $F_{i}$ is the receptive field of the i_th layer, k is the size of the convolutional kernel, L is the number of convolutional layers in each residual block, and $d_{i}$ is the dilation factor of the i_th layer.

**Figure 5:**
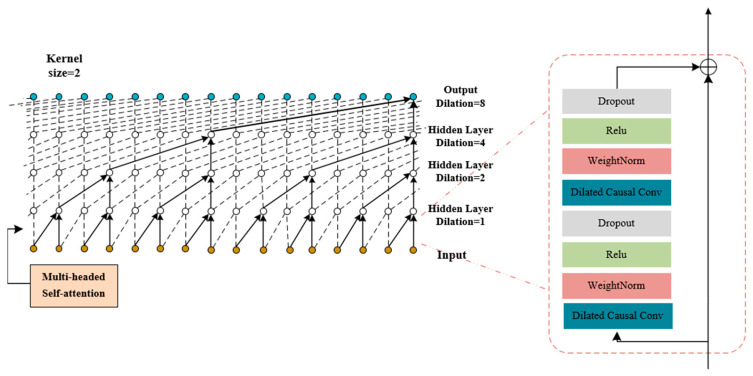
Structure diagram of MHSA-TCN.

To make the network work better, we have added a complex feature called multi-head self-attention mechanism (MHSA) that we discussed in Section 3.2.2 to TCN. Look at Figure 5 to see how MHSA-TCN is structured.

The MHSA-TCN module consists of the Multi-Head Self-Attention (MHSA) mechanism and the Temporal Convolutional Network (TCN). Specifically, the MHSA is integrated between the first and second hidden layers of the TCN, enabling the model to attend to different aspects of the input sequence. This network takes the local features it already obtained from MHSA-CRNN and SA-CRNN to find global EEG features. This model first performs downsampling on the input, where the scaling factor indicates how many time steps are skipped for sampling in each layer. Subsequently, the sampled data undergo weight normalization and Dropout operations. Finally, the processed signal is added to the original signal to generate the output. In this experiment, the TCN comprises four hidden layers, with each hidden layer incorporating two overlapping operations as described above, and each hidden layer having 25 channels, the kernel size = 2, and dilation factors of d = [1,2,4,8].

## 4. Experiments

### 4.1. Datasets

The DEAP dataset [46], also known as the Database for Emotion Analysis using Physiological Signals, is a comprehensive collection of data utilized by researchers to gain a deeper understanding of emotions. This dataset was compiled by researchers at the Queen Mary University of London and encompasses various physiological signals from the body and brain of 32 individuals, consisting of an equal number of male and female participants with an average age of 26 years. Additionally, videos capturing the facial expressions of 22 participants were recorded to observe their reactions. The research involved having the participants watch short music videos and evaluate their emotional responses. Parameters such as arousal level, valence, liking or disliking, dominance, and familiarity were measured using the Self-Assessment Manikin (SAM) scale. Notably, each participant viewed 40 different videos in real-time to ensure the accuracy of data collection. The ratings were based on a two-dimensional arousal–valence model rated on a scale from 1 to 9. The DEAP dataset includes 32 channels of EEG signals and 8 channels of peripheral physiological signals, all recorded at a rate of 512 Hz. 

For this experiment, we decided to focus solely on the EEG signals for emotion recognition, ditching the peripheral data. The experimental data within the DEAP dataset is split into 32 separate files, each representing an individual experimental subject. Within each participant’s file, you can find two arrays of data, which, after some basic preprocessing, are shown in Table 3.

The SEED dataset is an EEG dataset developed by Shanghai Jiao Tong University. This dataset consists of 62-channel EEG recordings from 15 subjects (7 males and 8 females, aged 23.27 ± 2.37 years) using the international 10–20 system. The subjects’ emotions were elicited through 15 video clips, each lasting 4 min. It measures three types of emotions (positive, neutral, and negative), with each emotion associated with five video clips. During playback, videos of the same emotion type do not appear consecutively but instead, videos of different emotions alternate. After watching the film clips, each subject was subjected to self-assessment.

### 4.2. Data Preprocessing 

#### 4.2.1. Data Preprocessing for the DEAP Dataset

When processing EEG data from the DEAP dataset, we first downsampled the signals to 128 Hz. Then, the 63 s data were segmented into 1-s windows, and the arithmetic mean of the first three 1 s baseline segments was calculated. Subsequently, the mean baseline was subtracted from each of the remaining 60 segments to remove the baseline. The calculation process is as follows:

(9) $$s_{baseline}=\;\frac{\sum_{i=1}^{3}x_{i}}{3}$$

(10) $$s=s_{original}-s_{baseline}$$

where $s_{baseline}$ represents the average baseline signal, $s_{original}$ is the original EEG signal, and s represents the signal after baseline removal. Finally, the 60 1 s segments after baseline removal are rearranged in chronological order into 3 s segments as input for the model.

Similar to the literature [47], this experiment adopts two schemes based on the valence dimension and arousal dimension for the processing of emotional labels. The first uses 5 as the threshold, arousal and valence SAM scores 1–5 are classified as low scores, 6–9 as high scores, low (LA) and high (HA) in the arousal dimension, and low (LV) and high (HV) in the valence dimension. In the second scheme, 4 and 6 were used as thresholds. Arousal and valence SAM scores of 1–4 were classified as low scores, 5–6 as medium scores, 7–9 as high scores, low (LA), medium arousal (MA), and high (HA) in arousal dimension, and low (LV), medium valence (MV), and high (HV) in valence dimension. The specific division of the two schemes is shown in Table 4.

#### 4.2.2. Data Preprocessing for the SEED Dataset

When processing EEG data from the SEED dataset, we employed similar data preprocessing methods as those utilized for the DEAP dataset. Initially, we downsampled the signals to 128 Hz and then segmented them using a 1 s window. Subsequently, we arranged the 1 s segments in chronological order into 3 s segments as inputs for the model. In contrast to the DEAP dataset, the SEED dataset lacks baseline signals; therefore, there is no need for an operation to remove the baseline.

### 4.3. Experimental Setup

The DEAP dataset and SEED dataset were utilized to train and validate the model on a powerful GeForce RTX 2080 Ti GPU using the Pytorch version 1.9.5 framework in the research. The model was optimized using Adam as the optimizer, with a learning rate of 0.001, a batch size of 128, and a cross-entropy loss function. The data were split into a training set and a test set in an 8:2 ratio to ensure robust evaluation. To further evaluate our model’s performance, a rigorous 10-fold cross-validation approach was employed.

## 5. Results and Discussion 

### 5.1. Convergence of the Model

**Figure 6:**
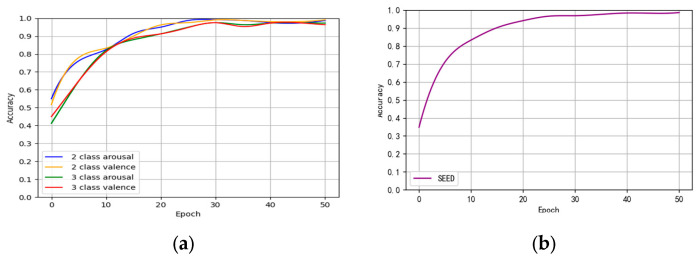
Accuracy–epoch relationship diagram. (**a**) DEAP dataset; (**b**) SEED dataset.

Figure 6a illustrates the fluctuating pattern of accuracy in binary and triple classification across the DEAP dataset during training. As the process unfolds, the accuracy experiences a steady increase before eventually stabilizing at a consistent level, highlighting the model’s strong convergence after a certain number of iterations. Notably, the data suggest that the model reaches its peak performance at around 30 iterations, demonstrating remarkable accuracy while utilizing minimal resources. Consequently, for this experiment, an epoch of 30 is considered ideal for optimal results.

Figure 6b illustrates the fluctuation pattern of accuracy during the training process on the SEED dataset. The accuracy shows a steady increase and eventually stabilizes at a consistent level, indicating strong convergence of the model after a certain number of iterations. It is worth noting that the data suggest that the model achieves its peak performance at approximately 40 iterations, demonstrating exceptional accuracy while utilizing minimal resources. Therefore, for experiments on the SEED dataset, 40 epochs are considered to yield the most optimal outcome.

### 5.2. Overall Performance

#### 5.2.1. Overall Performance on the DEAP Dataset

**Figure 7:**
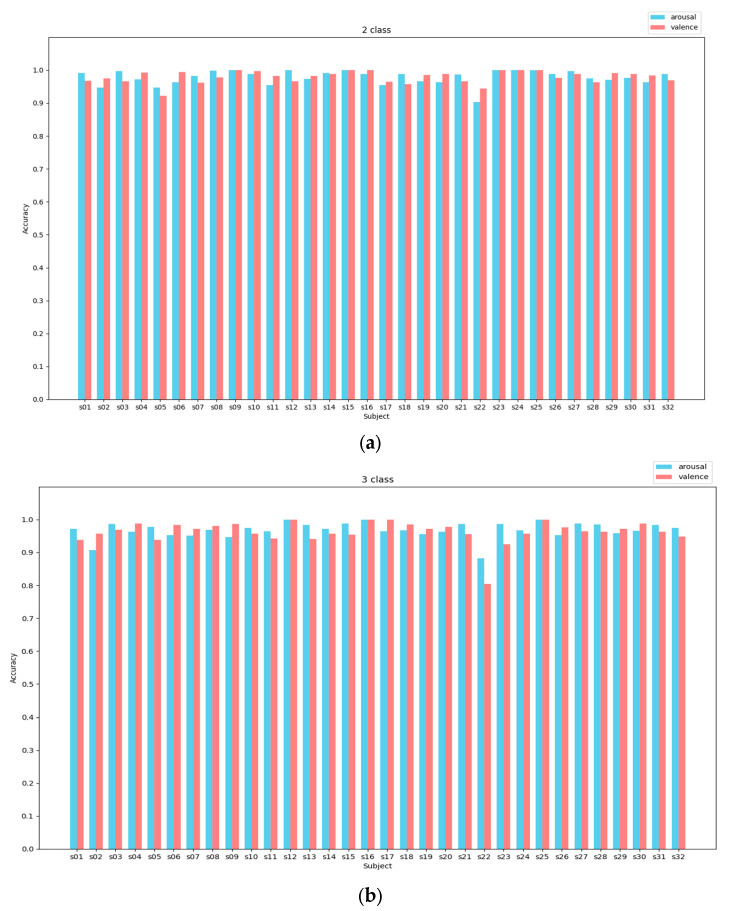
The average accuracy of arousal and valence of DEAP using CSA-SA-CRTNN for each subject. (**a**) 2-class (**b**) 3-class.

In Figure 6a, it is evident that the CSA-SA-CRTNN model is reliable and Effective when used on the DEAP dataset. Moving on to Figure 7, we can observe the accuracy of the model on individual subjects within the DEAP dataset, especially when the epoch is finely tuned to the ideal 30. 

In the binary experiment, the average accuracy for the valence dimension reached 99.15%, with the exceptions of s05 and s22, while for the arousal dimension, it reached 99.26%, except for s02 and s22. In the ternary classification experiment, the average accuracy of the valence dimension was 98.05% (except for s05 and s22), and in the arousal dimension, it was 97.69% (except for s02 and s22). It is noteworthy that subject No. 22 exhibited consistent characteristics in both two-category and three-category experiments, with significantly lower recognition accuracy compared to other subjects—achieving 90.27% and 93.75% in two-category valence and arousal, as well as 87.40% in three-category valence; whereas arousal achieved only 80.75% in three-category. This could be attributed to the possibility that the participants were distracted during the experiment or due to their subjective biases in scoring.

**Figure 8:**
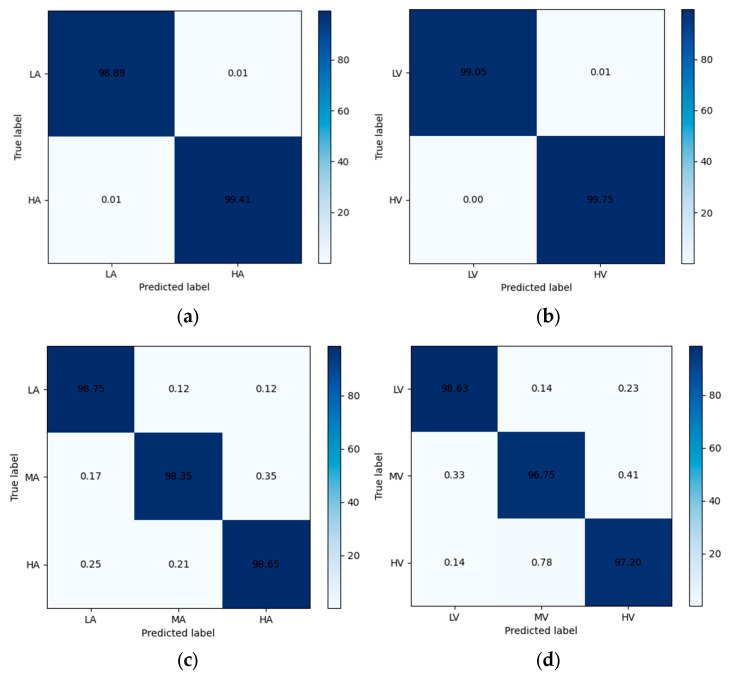
Confusion matrix: (**a**) 2-class arousal; (**b**) 2-class valence; (**c**) 3-class arousal; (**d**) 3-class valence.

The confusion matrix is shown in Figure 8. The confusion matrix shows the excellent classification ability of CSA-SA-CRTNN for each emotion category.

#### 5.2.2. Overall Performance on the SEED Dataset

**Figure 9:**
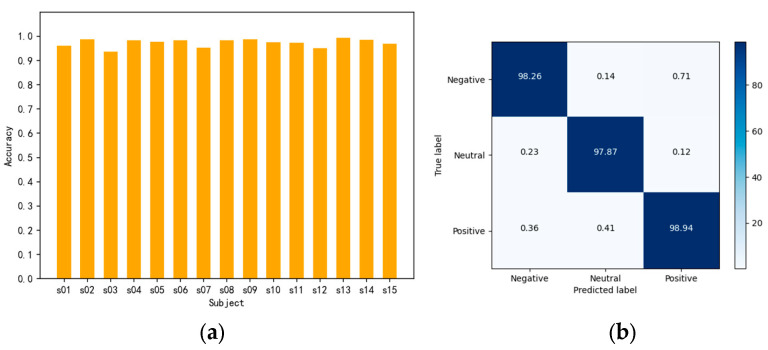
Experimental results on the SEED dataset: (**a**) Average accuracy for each subject; (**b**) confusion matrix.

From Figure 6b, it is observable that the CSA-SA-CRTNN model is reliable and effective when applied to the SEED dataset. Turning to Figure 9a, we can notice the accuracy of the model on individual subjects within the SEED dataset, particularly when the epoch is finely tuned to the ideal number of 40.

From Figure 9a, we can find that the accuracies of all subjects surpassed 95% and the average accuracy of them achieved 98.63% with STD 5.16%. Figure 9b shows the confusion matrix of the model on the SEED dataset, indicating that CSA-SA-CRTNN has excellent classification ability for each emotional category of the SEED dataset. 

### 5.3. Comparison with Related Research

To further evaluate the effectiveness of the model, we compared CSA-SA-CRTNN with previous studies on EEG-based emotional recognition. Our comparison methods are outlined below:

1. Tsception (Yi et al., 2021) [48]: Tsception is a cutting-edge deep learning model that can analyze EEG signals in a way that mimics the complexities of the human brain. It uses a dynamic temporal layer that can adapt to different time scales and frequencies present in EEG signals. Additionally, Tsception has an asymmetric spatial layer that takes into account the asymmetrical neural activations associated with emotional responses. This layer can pick up on subtle differences in how the brain processes information, allowing it to create a more accurate representation of the data it receives. Finally, Tsception has a fusion layer that brings together all the information;
2. DCRNN (Li et al., 2022) [49]: DCRNN sifts through EEG signals using a deep sparse autoencoder network (DSAE). Its mission is to cut through the noise and reconstruct the underlying features of EEG signals. By teaming up a convolutional neural network (CNN) with long short-term memory (LSTM), DCRNN digs deep into the connections between different parts of the brain and integrates contextual information from EEG signal frames;
3. 4D-CRNN (Shen et al., 2020) [50]: The 4D-CRNN is a groundbreaking technique that takes complex features from various sources and transforms them into an intricate, four-dimensional framework. It operates by leveraging the powers of both the convolutional neural network (CNN) and long short-term memory (LSTM) network. The CNN delves into the frequencies and spatial intricacies of each slice within the 4D input, while the LSTM uncovers the temporal relationships hidden within the CNN’s findings. The fusion of these two powerful networks results in a model that can truly understand and make sense of the intricate connections within the data;
4. BiSMSM(Li et al., 2023) [51]: BiSMSM is a complex framework that delves into the intricacies of time and space. It consists of two streams, one focusing on spatial aspects and the other on temporal aspects. Designed to decipher information from various viewpoints, including time, space, locality, and globality, this framework is made up of interconnected modules. The spatial and temporal streams mirror each other in structure, featuring a module centered around a multilayer perceptron (MLP). This module is key in unraveling both intra- and inter-channel insights from specific regions. Additionally, a self-attention mechanism module is in place to extract the global signal correlations;
5. AP-CapsNet (Liu et al., 2023) [52]: The AP-CapsNet method combines coordinated attention to help understand where things are about each other in the input data and then transforms this information into a complex space to identify emotions. To achieve this, a pre-trained model is utilized to extract features, and a double-layer capsule network is built for in-depth analysis;
6. ATDD-LSTM (Du et al., 2021) [53]: The ATDD-LSTM model functions as a mechanism to focus on specific segments of information produced by the LSTM to gain a deeper understanding of emotions. Additionally, it incorporates a domain discriminator to ensure consistency of information across diverse contexts;
7. ICaps-ResLSTM (Fan et al., 2024) [54]: ICaps-ResLSTM can understand the different patterns and locations within EEG data using capsule networks. In addition, it also has a ResLSTM module that helps it learn even more detailed and complex features by connecting different modules in time and space. This means it can pick up on even the most subtle differences in EEG data, making it good at distinguishing between different brain activities;
8. CADD-DCCNN (Li et al., 2024) [55]: To gain a deeper understanding of the emotions captured in EEG signals, utilizing DE features obtained through STFT. Each DE feature channel provides a unique perspective and employs an attention mechanism to extract key emotional data across the EEG timeline. CADD-DCCNN explores intricate interconnections among diverse timeframes, enhancing the comprehension of nonlinear relationships. Additionally, CADD-DCCNN incorporates a domain discriminator to ensure consistency in data representation and bridge gaps among different sources;
9. MDCNAResnet-BiGRU (Du et al., 2023) [56]: This method extracts differential entropy features from EEG signals and constructs a three-dimensional feature matrix. It utilizes deformable convolution to extract high-level abstract features and combines MDCNAResnet with bidirectional gated recurrent units (BiGRUs) to achieve emotion recognition;
10. TSFFN (Sun et al., 2022) [57]: This method introduces a multi-channel EEG emotion recognition model based on parallel transformers and Three-dimensional (3D) Convolutional Neural Networks (3D-CNN). It involves creating parallel channel EEG data and reconstructing EEG sequence data using location information. Then, the model utilizes transformers and 3D-CNN to extract temporal and spatial features from EEG data;
11. GLFANet (Liu et al., 2022) [58]: This method utilizes the spatial positions of EEG signal channels and the frequency domain features of each channel to construct an undirected topological graph to represent the spatial connection relationship between channels. Then, GLFANet is used to learn the deeper features of the undirected topological graph for emotion recognition.

Table 5 presents a comparison of the outcomes yielded by these methods. As depicted in the table, CSA-SA-CRTNN attains an accuracy of 99.26% and 99.15%, respectively, on the DEAP dataset and an accuracy of 98.63% on the SEED dataset. In contrast to existing methods, our model acquires more competitive results.

### 5.4. Ablation Study

The proposed model consists of four modules: the CSAM module, the SA-CRNN module, the MHSA-CRNN module, and the MHSA-TCN module. To evaluate the contribution of each module in the model, we conducted four types of ablation experiments on the DEAP dataset.

Specifically, four ablation models were constructed: SA-CRTNN, CSA-CRTNN, CA-SA-CRTNN, and CSA-SA-CRNN. Among them, SA-CRTNN is the model that abandons the CSAM module. CSA-CRTNN is the model without the SA-CRNN module. CA-SA-CRTNN is a model excluding the MHSA-CRNN module. CSA-SA-CRNN is a model that removes the MHSA-TCN module, and the detailed composition of each model is shown in Table 6.

Each model was tested on the DEAP dataset for binary and ternary classification experiments, and the results are shown in Table 7. Currently, our proposed model achieves the best accuracy, with 99.26% and 99.15% for binary classification and 97.69% and 98.05% for ternary classification, respectively. Compared to the proposed model, SA-CRTNN has the largest decrease in accuracy, with 13.01% and 14.15% for binary classification and 13.95% and 19.30% for ternary classification, respectively. This indicates that the proposed channel attention module CSAM plays a crucial role in the model. Without CSAM, the model may focus on some useless information, leading to a decrease in accuracy. CSA-CRTNN has the second largest gap in accuracy compared to the proposed model, with 6.76% and 4.16% for binary classification and 3.94% and 11.80% for ternary classification, respectively. This suggests that the proposed SA-CRNN module can effectively address the issue of information omission during feature extraction. CSA-SA-CRNN ranks third in terms of accuracy gap with the proposed model, with 4.47% and 6.24% for binary classification and larger gaps of 10.61% and 9.51% for ternary classification, respectively. This underscores the necessity of global feature extraction. CA-SA-CRTNN has the smallest decrease in accuracy, with 2.11% and 2.90% for binary classification and 1.15% and 3.06% for ternary classification; this indicates that MHSA-CRNN has a certain contribution to the model.

### 5.5. CSAM Compared with Other Channel Attention Mechanisms

**Figure 10:**
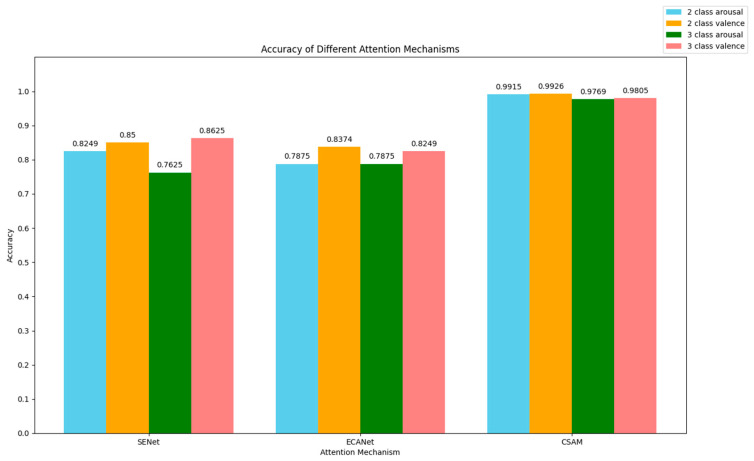
Comparison of attention mechanisms in different channels.

To validate the efficacy of the channel attention mechanism proposed in this paper, we conducted a comparative analysis with the commonly used mechanisms SENet and ECANet. The results are depicted in Figure 10.

SENet [59] gives each feature channel in a feature map a weight value. The network can concentrate on certain characteristics since the squeeze and excitation procedures automatically determine the significance of each channel. This method aids in enhancing the network’s performance across a range of jobs.

The ECANet neural network architecture [60] eliminates the requirement for fully connected layers by using 1 × 1 convolutional layers after the global average pooling layer. This method helps to prevent size reduction. In addition, the design achieves cross-channel information exchange by using dimensional convolution. The size of the convolutional kernel is adaptive, which means that layers with many channels can interact more across channels.

Based on the experimental results, it has been found that the channel attention mechanism, CSAM, proposed in this paper, outperforms SENet and ECANet. This suggests that in our model, CSAM can more accurately identify the important channels of EEG signals in comparison to other commonly used channel attention mechanisms. Consequently, this leads to an improvement in the recognition accuracy of the model. 

### 5.6. Evaluation of Model Efficiency

The primary objective of this paper is to enhance model accuracy while simultaneously reducing computational costs. To illustrate the capability of CSA-SA-CRTNN in reducing the computational burden of the model, we have presented the parameters of the proposed model in Table 8 and compared them with those of four other models in Table 9. The findings indicate that our model exhibits higher efficiency, with model parameters totaling 2.90 M. This outcome suggests that the model can achieve superior accuracy while consuming fewer resources.

### 5.7. Other Discussion

While the model has made significant improvements in accuracy, it is important to acknowledge its limitations. Firstly, despite its ability to capture more detailed temporal and spatial features, the complexity of the model structure presents a major obstacle in practical applications, making it challenging to meet the conditions for real-world deployment directly. Secondly, the recognition performance of the model is influenced by the configuration of experimental parameters, which adds to operational difficulties and results uncertainty. Furthermore, while the model demonstrates exceptional performance in independent dataset testing, its accuracy decreases in complex scenarios involving multi-person datasets due to significant individual differences.

In the future, our focus will be on optimizing the network structure, aiming for simplification to improve model operation efficiency. Furthermore, we will incorporate multi-modal data to strengthen the model’s generalization capabilities, effectively reducing the potential impact of individual differences on emotion recognition performance and ensuring a more precise and stable capture of emotional variations.

## 6. Conclusions

In this work, we introduce CSA-SA-CRTNN, a novel model for emotion recognition. To increase accuracy, our model is made to extract pertinent data from several viewpoints, such as temporal, spatial, local, and global. The CSAM channel attention module is introduced to improve the classification performance by assigning varying weights to distinct channels. Additionally, we introduce SA-CRNN, an adaptive convolutional recurrent module that tackles the problem of missing information during feature extraction. To extract local and global information, we also integrate CRNN and TCN with the multi-head self-attention mechanism, MHSA. We conducted binary and ternary classification experiments on the DEAP dataset, achieving 99.26% and 99.15% accuracy for arousal and valence in binary classification and 97.69% and 98.05% in ternary classification, and on the SEED dataset, we achieved an accuracy of 98.63%, demonstrating the effectiveness of our model. Furthermore, our model is efficient, achieving the highest accuracy with lower computational costs than other models. 

The experimental results demonstrate that the proposed model can effectively utilize EEG information from various aspects to enhance the classification accuracy of emotion recognition. Furthermore, this model significantly reduces computational costs and improves model efficiency, offering a more efficient and precise solution for EEG-based emotion recognition tasks.

## Figures and Tables

**Table 1:** Parameter settings of CSAM.

Layer	Parameter Settings	Activation Function
global average pool	kernel size = 32, stride = 1	—
Linear	Input = 32, Output = 64	—
Conv1D	kernel size = 3, stride = 1, padding = 1	Tanh
Conv1D	kernel size = 3, stride = 1, padding = 1	Tanh
Linear	Input = 64, Output = 32	Sigmoid

**Table 2:** Composition and parameter settings of CRNN.

Layer	Parameter Settings	Output
Conv	kernel size = (32,40), stride = 1	(384,40,1,89)
ELU	—	(384,40,1,89)
MaxPool	kernel size = (1,75), stride = 10	(384,40,1,2)
LSTM	input dimension = 80, hidden layer dimension	(128,3,64)

**Table 3:** DEAP dataset.

Array Name	Array Shape	Array Contents
data	40 × 40 × 8064	video/trial × channel × data
labels	40 × 4	video/trial × label (valence, arousal, dominance, liking)

**Table 4:** Data annotation scheme.

Task	Status	Emotion Label	Label Scores
2-class classification	Arousal	LA	1 ≤ A ≤ 5
		HA	5 < A ≤ 9
	Valence	LV	1 ≤ V ≤ 5
		HV	5 < V ≤ 9
3-class classification	Arousal	LA	1 ≤ A ≤ 4
		MA	4 < A ≤ 6
		HA	6 < A ≤ 9
	Valence	LV	1 ≤ V ≤ 4
		MV	4 < V ≤ 6
		HV	6 < V ≤ 9

**Table 5:** Performance comparison of different methods (Accuracy% ± Standard deviation%).

Method	DEAP		SEED
	Arousal	Valence	
Tsception (Yi et al., 2021) [48]	63.75 ± 11.04	62.27 ± 7.60	—
DCRNN (Li et al., 2022) [49]	81.43 ± 8.24	76.70 ± 13.07	—
4D-CRNN (Shen et al.,2020) [50]	94.58 ± 3.69	94.22 ± 3.69	94.74 ± 2.32
BiSMSM (Li et al., 2023) [51]	61.89 ± 6.60	63.10 ± 4.79	—
AP-CapsNet (Liu et al., 2023) [52]	95.04 ± 3.17	93.89 ± 8.63	—
ATDD-LSTM (Du et al., 2021) [53]	72.97 ± 6.57	69.06 ± 6.37	79:26 ± 12.79
ICaps-ResLSTM (Fan et al., 2024) [54]	98.06 ± 1.24	97.94 ± 1.32	—
CADD-DCCNN (Li et al., 2024) [55]	92.42 ± 12.72	90.97 ± 13.96	92.44 ± 06.16
MDCNAResnet-BiGRU (Du et al., 2023) [56]	98.89 ± 1.22	98.63 ± 1.51	98.13 ± —
TSFFN (Sun et al., 2022) [57]	98.53 ± —	98.27 ± —	97.64 ± —
GLFANet (Liu et al., 2022) [58]	94.91 ± —	94.53 ± —	93.19 ± —
**CSA-SA-CRTNN(Ours)**	**99.26 ± 7.09**	**99.15 ± 6.85**	**98.63 ± 5.16**

**Table 6:** Ablation model.

Model	Module			
	CSAM	SA-CRNN	MHSA-CRNN	MHSA-TCN
SA-CRTNN	×	√	√	√
CSA-CRTNN	√	×	√	√
CA-SA-CRTNN	√	√	×	√
CSA-SA-CRNN	√	√	√	×

**Table 7:** Ablation study results of CSA-SA-CRTNN on the DEAP dataset.

Model	Accuracy (%) ± Standard Deviation (%)			
	2 Class		3 Class	
	Arousal	Valence	Arousal	Valence
SA-CRTNN	86.25 ± 0.29	85.00 ± 0.78	83.74 ± 0.32	78.75 ± 0.47
CSA-CRTNN	92.50 ± 3.15	94.99 ± 4.95	93.75 ± 3.61	86.25 ± 2.13
CA-SA-CRTNN	97.15 ± 4.89	96.25 ± 4.44	96.54 ± 3.82	94.99 ± 5.15
CSA-SA-CRNN	94.79 ± 2.78	92.91 ± 4.10	87.08 ± 3.27	88.54 ± 2.17
**CSA-SA-CRTNN(Ours)**	**99.26 ± 7.09**	**99.15 ± 6.85**	**97.69 ± 10.70**	**98.05 ± 5.39**

**Table 8:** Parameters of the CSA-SA-CRTNN model.

Name	Output Size	Params Size
CSAM	(128,3,32,128)	16.5 K
MHSA-CRNN	(128,3,64)	285.7 K
SA-CRNN	(128,3,64)	285.2 K
MHSA-TCN	(128,25,64)	138 K
Fully connected	(128,25,64)	130
Trainable params	-	726 K
Total model params	-	2.90 M

**Table 9:** Comparison of the efficiency of different models.

Model	Params	Accuracy (%)	
		Arousal	Valence
HSLT (Wang et al., 2022) [61]	30.6 M	66.20	66.63
TH-FM (Topic et al., 2021) [62]	39.07 M	75.44	74.91
TDMNN (Ju et al., 2024) [63]	15.25 M	98.25	98.08
ATCapsLSTM (Deng et al., 2021) [64]	28.92 M	97.17	97.34
**CSA-SA-CRTNN(Ours)**	**2.90 M**	**99.26**	**99.15**

## Data Availability

The database used in this study is publicly available at the following websites: DEAP—http://www.eecs.qmul.ac.uk/mmv/datasets/deap/ (accessed on 25 November 2023).

## Abbreviations

The following abbreviations are used in this manuscript:

EEG	electroencephalogram
DEAP	Dataset for Emotion Analysis using Physiological Signals
CNN	Convolutional Neural Networks
SVM	Support Vector Machines
KNN	K-nearest neighbors
CapsNet	capsule networks
LSTM	long short-term memory networks
LR	Lattice Reduction
DBN	Deep Belief Networks
SEED	SJTU Emotion EEG Dataset
CRNN	Convolutional Recurrent Neural Network
RNN	Recurrent Neural Network
GCN	Graph Convolutional Networks
XGBoost	eXtreme Gradient Boosting
ResNet	Residual Network
TCN	Temporal Convolutional Networks
HV	High Valence
HA	High Arousal
HV	High Valence
LA	Low Arousal
MV	Medium Valence
MA	Medium Arousal
DSAE	Deep Sparse Autoencoder Network
MLP	Multilayer
BiGRUs	Bidirectional Gated Recurrent Units
SENet	Squeeze-and-Excitation Networks
ECANet	Efficient Channel Attention Network
